# Incorporation of PEG Diacrylates (PEGDA) Generates Hybrid Fmoc-FF Hydrogel Matrices

**DOI:** 10.3390/gels8120831

**Published:** 2022-12-16

**Authors:** Elisabetta Rosa, Enrico Gallo, Teresa Sibillano, Cinzia Giannini, Serena Rizzuti, Eliana Gianolio, Pasqualina Liana Scognamiglio, Giancarlo Morelli, Antonella Accardo, Carlo Diaferia

**Affiliations:** 1 Department of Pharmacy, Research Centre on Bioactive Peptides (CIRPeB), University of Naples “Federico II”, Via Montesano 49, 80131 Naples, Italy; 2 IRCCS Synlab SDN, Via E. Gianturco 113, 80143 Naples, Italy; 3 Institute of Crystallography (IC), CNR, Via Amendola 122, 70126 Bari, Italy; 4 Department of Molecular Biotechnologies and Health Science, University of Turin, Via Nizza 52, 10125 Turin, Italy; 5 Department of Sciences, University of Basilicata, Via dell’Ateneo Lucano 10, 85100 Potenza, Italy

**Keywords:** Fmoc-FF, PEGDA, peptide hydrogels, supramolecular assembly, peptide materials, multicomponent hydrogels, biohybrid materials

## Abstract

Generated by a hierarchical and multiscale self-assembling phenomenon, peptide-based hydrogels (HGs) are soft materials useful for a variety of applications. Short and ultra-short peptides are intriguing building blocks for hydrogel fabrication. These matrices can also be obtained by mixing low-molecular-weight peptides with other chemical entities (e.g., polymers, other peptides). The combination of two or more constituents opens the door to the development of hybrid systems with tunable mechanical properties and unexpected biofunctionalities or morphologies. For this scope, the formulation, the multiscale analysis, and the supramolecular characterization of novel hybrid peptide-polymer hydrogels are herein described. The proposed matrices contain the Fmoc-FF (N^α^-fluorenylmethyloxycarbonyl diphenylalanine) hydrogelator at a concentration of 0.5 wt% (5.0 mg/mL) and a diacrylate α-/ω-substituted polyethylene-glycol derivative (PEGDA). Two PEGDA derivatives, PEGDA 1 and PEGDA2 (mean molecular weights of 575 and 250 Da, respectively), are mixed with Fmoc-FF at different ratios (Fmoc-FF/PEGDA at 1/1, 1/2, 1/5, 1/10 mol/mol). All the multicomponent hybrid peptide-polymer hydrogels are scrutinized with a large panel of analytical techniques (including proton relaxometry, FTIR, WAXS, rheometry, and scanning electronic microscopy). The matrices were found to be able to generate mechanical responses in the 2–8 kPa range, producing a panel of tunable materials with the same chemical composition. The release of a model drug (Naphthol Yellow S) is reported too. The tunable features, the different topologies, and the versatility of the proposed materials open the door to the development of tools for different applicative areas, including diagnostics, liquid biopsies and responsive materials. The incorporation of a diacrylate function also suggests the possible development of interpenetrating networks upon cross-linking reactions. All the collected data allow a mutual comparison between the different matrices, thus confirming the significance of the hybrid peptide/polymer-based methodology as a strategy for the design of innovative materials.

## 1. Introduction

Hydrogels (HGs) are hydrophilic materials structurally characterized by a three-dimensional (3D) network originated by the macroscopic organization of polymer chains or of supramolecular elements [1,2,3]. The ability of these matrices to retain a large amount of aqueous fluid (~95/99%) confers on them a non-Newtonian flow behavior associated with a self-supporting tendency. The physicochemical properties of hydrogels (e.g., durability, reproducibility, and bio-restorability) and their resemblance to the human body tissue microenvironment were found to be determinant features for their application in both the biotechnological and industrial fields, including drug delivery [4], optoelectronics [5], water purification [6], and tissue engineering [7].

Peptide-based low-molecular-weight gelators (LMWGs) are a typical class of molecular entities that can be used for the generation of self-supporting three-dimensional gel networks [8,9,10]. The interest in this class of LMWGs is related to the advantages they offer with respect to natural or synthetic polymers. For instance, peptides can easily be synthetized and modified; moreover, they exhibit good biocompatibility profiles and moderately inexpensive manufacturing procedures. Additionally, in comparison to covalently-based polymeric hydrogels, the peptide gelation phenomenon is a multiscale process, which allows one to avoid crosslinking agents. Specifically, peptide-based LMWGs are designed as self-assembling sequences able to produce fibrillary aggregates [11,12,13]. These latter, above a critical gelation concentration (CGC), undergo a mutual physical cross-link. The final non-covalent entanglement leads to further association in a space-spanning network, giving rise to the macroscopic self-supporting gels.

**Figure 1:**
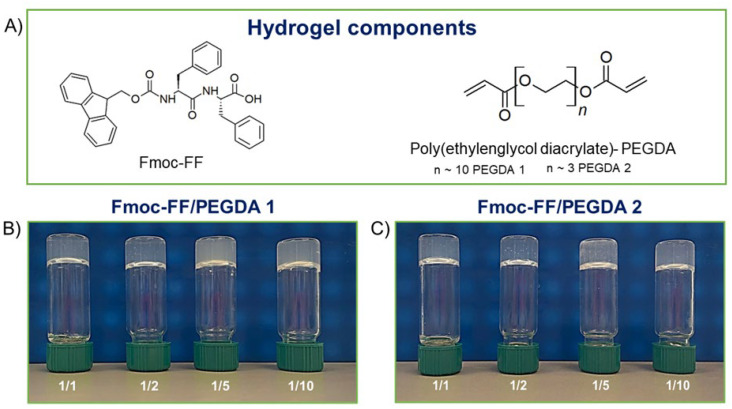
(**A**) Chemical structures of hydrogel components. Inverted tube test for mixed hydrogel formulations (0.5 wt% in Fmoc-FF) with PEGDA1 (**B**) and PEGDA2 (**C**).

Fmoc-FF (N^α^-fluorenylmethoxycarbonyl-diphenylalanine, Figure 1A) represents a typical case of peptide-based LMWGs [14,15,16,17,18,19]. HGs of Fmoc-FF can be obtained under physiological conditions (pH, temperature, and ionic strength) without the use of cross-linking agents by using different methodologies [20]. Since its identification in 2006 [14,15] as a suitable building block for the preparation of hydrogels, Fmoc-FF has been deeply studied for many biomedical applications [21,22,23,24]. The addition of other components (like natural or synthetic polymers [25], peptides [26,27], polysaccharides [28], or dyes [29]) to Fmoc-FF has been found to improve and tune the physiochemical features of the final material.

At the state of the art, co-assembly of two different chemical entities can sponsor the formation of novel functional materials with improved mechanical or biocompatibility profiles [30,31,32]. By way of examples, Fmoc-FF/pentafluorinate Fmoc-F [33] and Fmoc-FF/Phe-Tyr-containing peptides [34] systems have been recently formulated and proposed as innovative all-peptidic scaffolds for both tissue regeneration and drug delivery applications. The incorporation of different kinds of polymers was also estimated by studying hybrid Fmoc-FF-based matrices containing chitosan, polyethylene-glycol (PEG) [35], or polyaniline (PAni) [36]. Specifically, PEG and its α- and/or ω-substituted derivatives are particularly interesting due to their biocompatibility and pharmaceutical and biomedical advantages, including the increase in solubility and the in vivo protection of bioactive macromolecules, antibodies, and oligonucleotides [37,38,39,40].

After FDA approval, PEGylation has indeed become the method of choice for the delivery of biopharmaceuticals. For this, here is described the formulation and the supramolecular architecture of multicomponent hydrogels containing some derivatives of polyethylene glycol, the PEG diacrylates (PEGDA, Figure 1A), into the gelled Fmoc-FF matrix to expand the class of hydrogels as biotechnological tools. The advantage of using PEGDA derivatives is the presence in their formula of a diacrylate moiety that, according to opportune synthetic protocols, can undergo a controlled polymerization reaction.

For this study, two PEGDAs with different mean molecular weights (PEGDA 575 and PEGDA 250, named PEGDA1 and PEGDA2, respectively) were selected. Additionally, different molar ratios between Fmoc-FF and PEGDA derivatives (1/1, 1/2, 1/5, and 1/10 mol/mol) were studied with the aim of analyzing the effect of both molecular weight and relative polymer abundance on the organization and on the properties of the final hybrid matrix. The gelation process was prompted using the DMSO/H_2_O solvent switch methodology, incorporating PEGDA in the hydrogels during the organic solvent rehydration step. The water behavior in all the matrices was scrutinized, reporting the swelling ratio, the Langmuir frequency, the dehydration curves, and the relaxometric properties. Secondary structuration analysis was conducted recurring to fluorescence, circular dichroism (CD), FTIR spectroscopies, and wide-angle X-ray scattering (WAXS). Moreover, the mechanical properties of hybrid matrices were studied in rheological experiments, and their capability to encapsulate and release a drug was also preliminary evaluated using a fluorescent dye (Naphthol Yellow S) as a model.

## 2. Results and Discussion

### 2.1. Fmoc-FF/PEGDA Matrices Formulation

The Fmoc-N^α^-protected variant of diphenylalanine (FF), Fmoc-FF, represents one of the most studied ultra-short peptide sequences able to efficiently self-organize into β-sheet nanostructured fibrous hydrogels in physiological conditions [14,15]. Due to their easy preparation and long shelf-stability, Fmoc-FF matrices have been proposed as tools for the development of hybrid systems too, thus allowing enlargement of the available biomaterials for nanotechnology applications.

Fmoc-FF HGs can be produced using different approaches: solvent switch, pH switch, and the enzymatic method [20]. Due to the hydrophilic nature of the PEGDA components, the formulation of hybrid Fmoc-FF/PEGDA HGs at 0.5 wt% (5.0 mg/mL) was achieved using the solvent switch procedure. The Fmoc-FF concentration chosen for the present study was 0.5 wt% because it is above the critical gelation concentration (CGC) and is one of the most studied concentrations in literature [14,15,34]. According to the solvent-switch method, Fmoc-FF was primarily dissolved in DMSO as the organic phase at a concentration of 100 mg/mL. The resulting solution was then diluted in water (an antisolvent) to the desired final gel concentration. The rehydration triggers gel formation, and a vertexing step promotes efficient mixing of the solvent/antisolvent phases, ensuring sample homogeneity. For mixed Fmoc-FF/PEGDA HGs, the solvent switch procedure was modified to use PEGDA solution instead of water to trigger the hydrogel formation during the DMSO hydration phase. This methodology allows the Fmoc-FF matrix enrichment of polymer with different molar ratios with respect to the peptide component (9.53·10*^−^*^6^ mol) by preparing different PEGDA solutions. The Fmoc-FF/PEGDA ratios selected for the study are 1/1, 1/2, 1/5, and 1/10 mol/mol. In all the tested ratios, we can produce self-supporting materials, as testified by the inverted tube test in Figure 1B,C.

The maximum hydrogel ratio formation for PEGDA 1 was found for a 1/50 ratio (see Figure S1). On the contrary, no hydrogel formation was observed for PEGDA2 above a ratio of 1/10, indicating the impossibility of forming stable and reproducible PEGDA2 solutions. Fmoc-FF represents a key structural element for the formation of the proposed mixed matrices. Indeed, at the tested concentrations, both PEGDA derivatives are not able to gel alone. Fmoc-FF dipeptide, driving the initial aggregation process, probably allows the formation of PEGDA hydrogels at a concentration value lower than their CGC. As previously reported for pure or multicomponent HGs based on Fmoc-FF, the formation of the self-supporting matrix is associated with an opaque-to-limpid macroscopic transition. This change in optical transparency is attributed to the progressive fibril growth from spherulitic architectures acting as nucleation points [41]. The same macroscopic behavior was observed for all the tested Fmoc-FF/PEGDA samples. Moreover, no significant variations were detected in the gelation kinetics of multicomponent hydrogels of both PEGDA derivatives (at 1/1, 1/2 and 1/5 ratio) with respect to pure Fmoc-FF (~3 min). A slight increase in the gelation time (~1 min plus) is only detected for 1/10 PEGDAs ratio. As clearly shown in Figure 1, samples differ in turbidity. Whilst relatively translucent matrices are formed for 1/1 and 1/2 ratios, both PEGDA-containing HGs at 1/5 and 1/10 ratios are opaque. The macroscopic evidence of turbidity is quantified by UV-Vis measurements, looking at the absorbance values at 600 nm. At this wavelength, the light absorption from peptide chromophores is absent, and absorbance values can be ascribed to matrix turbidity, arising from scattering phenomena [42]. The absorbance values for Fmoc-FF/PEGDA 1 increase from 0.183 to 0.270 a.u. by passing from the ratio of 1/1 to 1/10 one. A similar behavior was also observed for PEGDA 2 containing HGs, but with a slightly lower increase in turbidity (from 0.166 to 0.224 a.u. from 1/1 and 1/10 ratios). The aging of HGs was evaluated over 6 months, keeping the material inverted. All the samples showed very good shelf stability without any macroscopic change in terms of homogeneity, visual appearance, and self-supporting behavior. Only a modest syneresis event (weight lost ~5%) was found for 1/10 ratio samples.

### 2.2. Water Behavior in Multicomponent HGs

Water represents the main component in gelled matrices, and it can be classified as strongly bound, weekly bound, and free (non-bound) water [43]. The analysis of the water behavior can provide valuable information about the supramolecular organization, permeation properties, pore architecture, and solute diffusion. The water behavior in the proposed multicomponent matrices was characterized using several macroscopic assays (swelling ratio, stability test longitudinal relaxation rate (R1 = 1/T1) as a function of the applied magnetic field.

The swelling ratio q at room temperature of each mixed HG was established as the percentage difference between the weight before and after overnight gel incubation in water. Values of q, reported in the Table 1, increase with the amount of PEGDA in the hybrid materials and with respect to pure Fmoc-FF (*q* = 29.7%). Moreover, q values for PEGDA1 were found to be higher than those of the corresponding PEGDA 2 containing matrices. This difference can probably be attributed to the more hydrophilic nature of PEGDA1 polymer respect to PEGDA2. Indeed, the higher number of ethoxy repetitions in PEGDA1 (around 3-fold higher) with respect to PEGDA2 could cause an increase in the number of H-bonding acceptor groups, thus expanding the interactions with water and consequently the swelling properties.

Analogously, the stability ratios (Δ*W*) were found depending on the PEGDA incorporation amount (see Table 1). The matrices’ stability was tested using a Ringer’s solution, which mimics the physiological ionic strength. As clearly shown by Δ*W* values, all the Fmoc-FF/PEGDA hydrogels are more stable than the pure Fmoc-FF matrix, which has a Δ*W* = 27.4% in the same experimental conditions.

The stability of hybrid hydrogels seems to improve with the increasing percentage of polymer in the gel, thus indicating that the presence of PEG diacrylate in the formulation preserves the materials from degradation. This major stability could also be attributed to the higher rigidity of mixed hydrogels with respect to the pure Fmoc-FF one (vide intra, rheological section). PEGDA incorporation additionally confers on matrices a functional property of water retention, which is evaluated by studying the dehydration phenomenon of both PEGDA at 1/1 and 1/10 mol/mol. As clearly visible in Figure S2, PEGDA-containing HGs were found to be able to hold back elevated percentages (>92%) of entrapped water with respect to pure Fmoc-FF matrices (<9%). This feature was found to be substantially independent from both PEGDA molecular weight and amount. The unexpected behavior related to water retention can be imputed to the hydrophilic nature of PEGDA, which is able to avoid the evaporation of water by keeping it strongly anchored via non-covalent interactions.

Finally, water dynamics in the hydrogel matrix have been investigated using a relaxometric approach. The analysis and quantification of the dynamic parameters related to water motion have often been investigated through the measurement of the applied magnetic field dependence of the relaxation rate (nuclear magnetic resonance dispersion (NMRD) profiles) of different water-containing materials [44,45,46,47,48,49]. Fitting of the experimental data in the proton frequency range between 0.01 MHz and 10 MHz allows one to distinguish between slower and faster motions, respectively, associated with water molecules constrained in the hydrogel scaffold or more freely diffusing water. 

**Figure 2:**
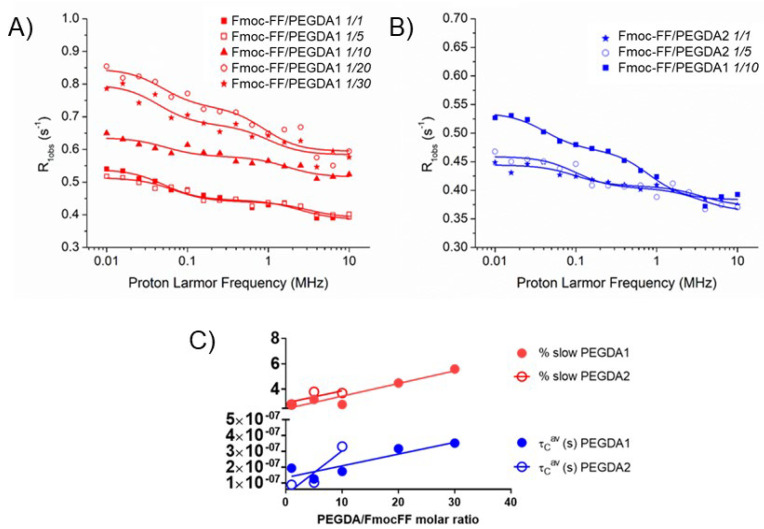
^1^H NMRD profiles measured on HGs with PEGDA1 (**A**) and PEGDA2 (**B**) at 298 K. Continuous lines represent the best fits obtained with a sum of Lorentzian functions. (**C**) Variation of the average water correlation time (τ_c_^aυ^) and the percentage of slowing moving water (% slow) as a function of PEGDA molar content.

In Figure 2A,B, the NMRD profiles acquired for Fmoc-FF/PEGDA HGs are reported. In the case of PEGDA1 containing HGs, two additional samples with higher polymer contents (1/20 and 1/30) were investigated. The data were fitted as previously reported, and the parameters extracted from fitting are collected in Table 2. In general, the here obtained τ1 (faster motion) and τ2 (slower motion) correlation times are rather similar to those previously observed for analogous Fmoc-FF-containing hybrid HGs [27]. Likewise, the percentage of slow motion (% slow) and the average correlation time (τ_C_^aν^), which can be calculated as previously achieved [27], are close to the previous values. For both PEGDA1 and PEGDA2, the values of % slow and τ_C_^aν^ (Table 2 and Figure 2C) increase with the amount of PEGDA in the hybrid materials but, quite surprisingly, are considerably lower with respect to pure Fmoc-FF. Moreover, the parameters associated with water mobility were found to be independent from PEGDA molecular weight.

### 2.3. Secondary Structure Characterization

It is well known from the literature that the Fmoc-FF peptide self-assembles into hydrogels with a β-sheet amyloid-like organization of the peptide moiety [14,15]. This structural motif is commonly investigated in peptide nanostructures using a combination of spectroscopic techniques (e.g., circular dichroism (CD) and Fourier transform infrared spectroscopy (FTIR)) and qualitative staining assays with Thioflavin T (ThT) and Congo Red (CR). The effect of PEGDA incorporation in the structural arrangement of the Fmoc-FF network was evaluated by comparing pure and hybrid peptide hydrogels.

**Figure 3:**
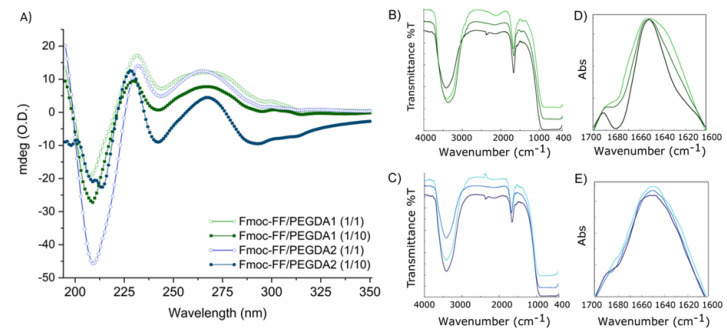
(**A**) CD spectra (in the 190 and 350 nm range) of Fmoc-FF/PEGDA1 (1/1 and 1/10) and Fmoc-FF/PEGDA2 (1/1 and 1/10). FTIR characterization. (**B**) FTIR spectra of Fmoc-FF/PEGDA1 matrices (1/1, light green; 1/5, green grass; and 1/10, dark green). (**C**) FTIR spectra of Fmoc-FF/PEGDA2 matrices (1/1, light blue; 1/5, blue; and 1/10, dark blue). Amide I deconvolution profiles of PEGDA1 (**D**) and PEGDA2 matrices (**E**).

CD spectra (Figure 3A) were acquired for Fmoc-FF/PEGDA hydrogels at 1/1 and 1/10 ratios between 350 and 190 nm and reported as optical density (mdeg/O.D.). The CD signature, deriving from the supramolecular packing of monomers, is related to the configurational alignment of the formed fibers, leading to higher order architectures. Independently from PEGDA molecular weight or their relative amounts, all the spectra show a similar signature, thus suggesting no significant differences in the gel organization and topography. Fmoc-FF/PEGDA systems present some differences in the dichroic signature with respect to the pure Fmoc-FF hydrogel alone [34], which exhibits a minimum at 220 nm and a broad maximum around 259 nm.

Indeed, the spectra of all the PEGDA-doped hydrogels showed four main dichroic signals: two negative peaks (around 209 and 240 nm) and two positive ones (at 231 and at 267 nm). These differences can be attributed to the presence of the PEG moiety since they were observed for other hybrid hydrogels containing Fmoc-FF in combination with PEG-peptide derivatives [34]. The signal located at 231 nm is generally indicative of β-sheet structuration of the peptide building block in supramolecular architectures [50,51,52]. This signal undergoes an hypsochromic effect (228 nm) in 1/10 matrices, imputable to the decreasing of the materials’ ability to absorb light because of the increased turbidity. Instead, the positive and broad band at 267 nm, detectable for all the mixed PEGDA hydrogels, can be attributed to the fluorenyl group on the peptide. The significant bathochromic effect at the typical wavelength (259 nm) can be explained considering the difference in the dielectric constant induced by the incorporation of PEGDA into the hybrid peptide-polymer matrices. The comparison with the CD profile of nude Fmoc-FF suggests a substantial maintenance of gel matrix topology without alteration of the β-sheet organization, with an antiparallel left-twisted structuration [53].

An additional investigation about the secondary structuration in the final material was carried out by FTIR spectroscopy. The IR spectra of peptides and proteins are described as containing nine different amide bands (I to VII, A and B). These IR signals are generated from both the vibrational contributions of the backbone and of amino acid side chains [54]. The IR spectra for all the matrices at 0.5 wt% in water are collected in Figure 3B,C. All the spectra share a common tendency, dominated by only two different bands: (i) an intense signal in the amide A region (~3300 cm^−1^), occurring as a consequence of water exposure of the aggregate with asymmetric and symmetric O-H and N-H stretching and indicative of intermolecular amide-amide bond interactions; (ii) a band in the amide I region (centered at 1636 cm^−1^) related to the presence of β-rich assemblies. This spectral region (ranging between 1700 and 1600 cm^−1^ associated with the C=O stretching motus) is of relevance for the secondary structuration analysis [55]. According to this consideration, an absorbance deconvolution was acquired (Figure 3D,E). For all the samples, the spectral deconvolution is dominated by a main peak around 1650 cm^−1^, conducive to C=O stretching and suggesting the presence of β-sheets secondary structures. Moreover, the additional band at ~1690 cm^−1^ is typically observed for the antiparallel orientation of the β-strands in assemblies [56,57,58].

**Figure 4:**
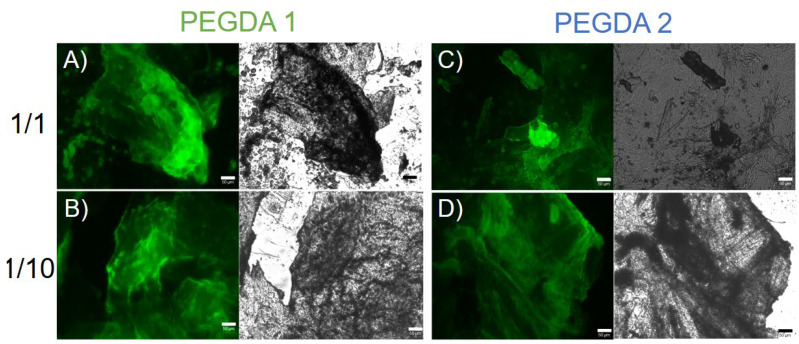
For each panel, fluorescence images (left) and optical images (right) of mixed xerogels (**A**) Fmoc-FF/PEGDA1 1/1, (**B**) Fmoc-FF/PEGDA1 1/10, (**C**) Fmoc-FF/PEGDA2 1/1 and (**D**) Fmoc-FF/PEGDA2 1/10 stained with 50 µmol/L ThT solution. Samples are imaged in the spectral regions of the GFP (Green Fluorescent Protein, λ_exc_ = 488 nm, λ_em_ = 507 nm) and in the bright field. Scale bar = 50 μm.

The common IR signature for all the matrices suggests that the fundamental β-secondary structure organization is not significantly altered by both the PEGDA length and the percentage of polymer incorporation, as supported by CD measurements. The formation of β-sheets rich matrices was further confirmed on xerogels by the thioflavin T (ThT) assay. Thioflavin T (ThT, alternatively named methylene yellow or Basic Yellow 1) is a benzothiazole fluorescent dye regularly used for the quantification of amyloid and amyloid-like fibrils. Binding amyloid-like structures, ThT changes its fluorescent behavior, with a strong fluorescence signal located approximately at 482 nm (λ_exc_ = 450 nm) [59,60]. The fluorescence activation of ThT is imputable to the rotational immobilization of the central C–C bond connecting the benzothiazole and aniline rings. As visible in Figure 4, the ThT dye can stain the PEGDA-containing HGs, giving rise to a fluorescent emission in the GFP spectral window. This evidence highlights the presence of an amyloid-like structure as a supramolecular element of the hydrogels.

**Figure 5:**
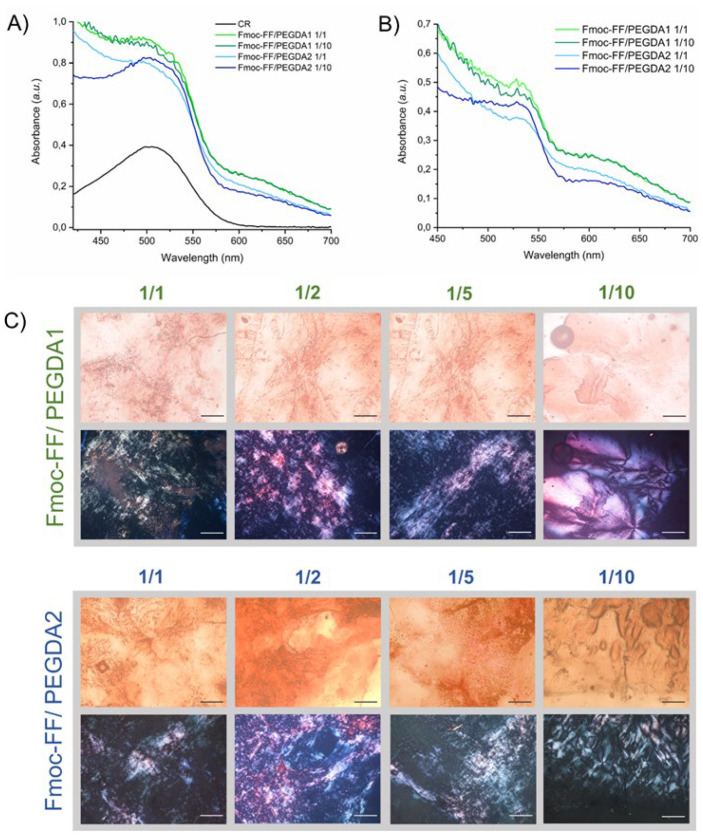
CR assay in solution and in the solid state. UV-Vis spectra of CR alone and co-incubated with Fmoc-FF/PEGDA1 (**A**) or with Fmoc-FF/PEGDA2 (**B**) at the different molar ratios. (**C**) Staining of Congo Red xerogels in both bright field (first row) and under cross-polarized light (second row). Scale bar =100 μm.

The presence of an amyloid-like structure was further confirmed using the Congo Red (sodium salt of benzidinediazo-bis-1-naphthylamine-4-sulfonic acid) assay, both on samples in solution and in the solid state (Figure 5).

The UV-Vis spectra of mixed hydrogels prepared in a CR solution at the final dye concentration of 10 µM are reported in Figure 5A,B. The spectrum of the dye alone in water is also reported for comparison. From the inspection of the figure, a red shift of the absorbance peak can be observed from 480 to 540 nm. This bathochromic shift of the maximum is typically associated with the detection of β-sheet secondary structures in the sample. Analogously, amyloid-like structures are also confirmed by optical microscopy images, under bright field and cross-polarized light, of mixed xerogels stained with CR (Figure 5C). Images showed that, independently from the composition and the PEGDA molecular weight, all the xerogels exhibit birefringence.

### 2.4. Solid State Characterization

**Figure 6:**
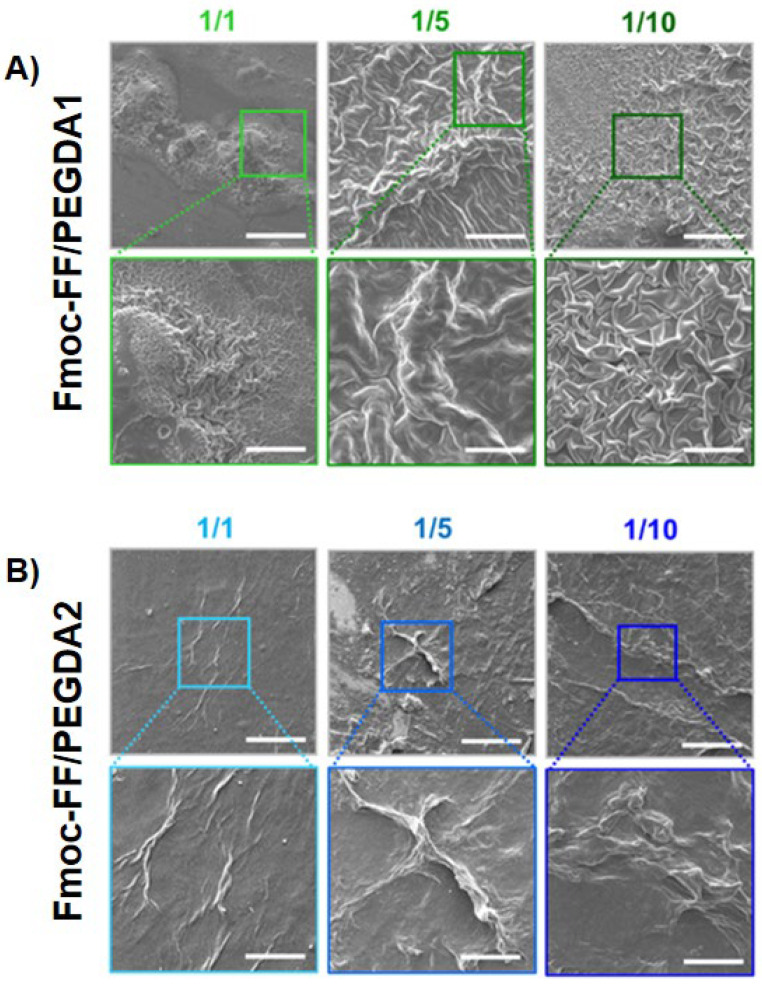
Selected microphotos of mixed xerogels of PEGDA1 (**A**) and PEGDA2 (**B**) series. Scale bar = 100 µm and 30 µm.

To collect information about the morphology of mixed xerogels, scanning electron microscopy (SEM) images were acquired. A set of representative microphotos for samples containing increasing amounts of diacrylate are shown in Figure 6. A substantial difference in the surface topography of systems can be detected with respect to the sponge-like structure (generally reported for PEGDA-based matrices) [61,62] and the entangled fiber network (noticed for HGs of Fmoc-FF) [14,15,34]. This primary evidence is indicative of a modification of the aggregation properties of both chemical building blocks, reinforcing the evidence that co-aggregation enlarges the plethora of supramolecular behaviors of molecules. More specifically, the PEGDA1 and PEGDA2 matrices mutually differ.

For PEGDA1 xerogels, a quasi-fractal drapery surface is detected. The geometric distribution, the fineness, and the grade of detail increase with the amount of PEGDA polymer, letting us postulate that the change in surface morphology is attributed to its intermolecular interaction. On the contrary, PEGDA2 hydrogels show fiber-like architectures, which manifest more in matrices with higher polymer ratios.

The evident discrepancies between PEGDA1 and the two samples indicate a rule of polymer molecular weight in the topological arrangements of the matrices, probably imputable to a different network of H-bonds. This specific physicochemical parameter also changed the peptide-polymer interaction networking, in turn producing very different superficial morphologies in hybrid matrices.

Further structural characterization of the peptide’s supramolecular architecture was achieved by a wide-angle X-ray scattering (WAXS) study. Measurements were collected on the macroscopic fibers of samples prepared according to the stretch-frame method [63].

**Figure 7:**
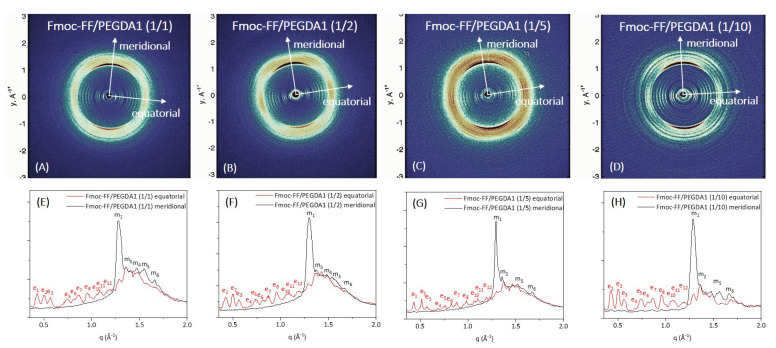
WAXS characterization of the mixed hydrogels Fmoc-FF/PEGDA1 at different concentrations: 2D WAXS data (on the top row right) and 1D WAXS meridional (black line)/equatorial (red line) profiles (on the bottom row).

**Figure 8:**
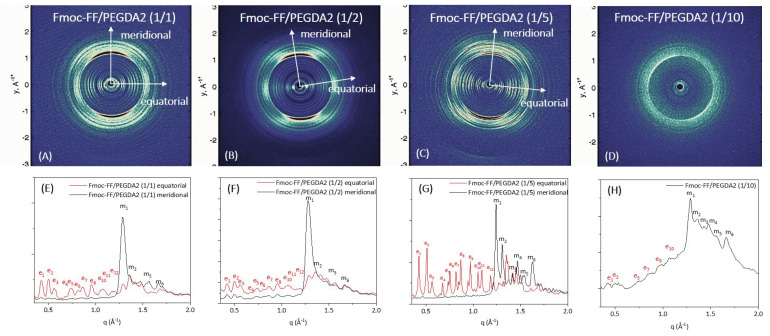
WAXS characterization of the mixed hydrogels Fmoc-FF/PEGDA2 at different concentrations: 2D WAXS data (on the top row right) and 1D WAXS meridional (black line)/equatorial (red line) (on the bottom row).

In all the cases, the WAXS data present the typical fiber diffraction pattern with two crossed main axes: the meridional along the fiber direction and the equatorial perpendicular to it (depicted by white arrows in 2D WAXS patterns). The 1D profiles, reported in Figure 7 and Figure 8E–H, have been obtained from the integration along both meridional and equatorial axes of 2D data, and the principal peaks along the axis are schematically summarized in Table S1.

In all the cases, the WAXS data present the typical fiber diffraction pattern with two crossed main axes: the meridional along the fiber direction and the equatorial perpendicular to it (depicted by white arrows in 2D WAXS patterns). As reported in Figure 7 and Figure 8E–H, 1D profiles have been obtained from the integration along both meridional and equatorial axes of 2D data, and the principal peaks along the axis are schematically summarized in Table S1. 

According to data previously collected on the Fmoc-FF hydrogel, hybrid gels containing PEGDA1 or PEGDA2 polymers exhibit the well-known diffraction pattern of cross-β amyloid-like structures, and the two axes (meridional and equatorial) correspond to the axis along the fiber and perpendicular to it, respectively [64]. The main diffraction peak at q = 1.29 Å^−1^ (d = 4.9 Å) gives information on the distance existing between adjacent peptide backbones organized into β-strands along the fiber axis. On the other hand, the peak at q ∼ 0.5 Å^−1^ (d = 12.5 Å) can be associated with the distance between two distinct β-sheets. The only exception is the absence of cross-β amyloid-like structures for the sample Fmoc-FF/PEGDA2 (1/10) due to the difficulty of realizing the solid fiber with the described method. The WAXS profiles of mixed hydrogels are very similar to those previously measured for pure Fmoc-FF, whose 2D and 1D WAXS results are reported in Figure S3.

The relevant difference we detect in mixed Fmoc-FF/PEGDA1 and Fmoc-FF/PEGDA2 hydrogels (at different ratios) with respect to pure Fmoc-FF is the presence of several additional equatorial and meridional reflections (see Figures S4 and S5 and Table S1 for positions and corresponding distances). This finding suggests a significant increase in the hierarchical order along the fiber induced by PEGDA1 and PEGDA2 (especially at the higher concentrations of the polymeric component).

### 2.5. Rheological Characterization

The mechanical response of matrices was evaluated via rheological analysis, describing the viscoelastic behavior of each Fmoc-FF/PEGDA hydrogel (at 0.5 wt%) in terms of G’ (storage modulus) and G’’ (loss modulus). The analysis was conducted by performing time-sweep oscillatory measurements (for 15 min, with 1.0 Hz frequency and 0.1% strain), supported by a preliminary identification of the optimal measurement conditions. Specifically, dynamic oscillation strain sweep (at a frequency of 1.0 Hz) and dynamic frequency sweep (at 0.1% strain) were acquired for Fmoc-FF/PEGDA at the two ratios of 1/1 and 1/10 (Figures S6 and S7).

**Figure 9:**
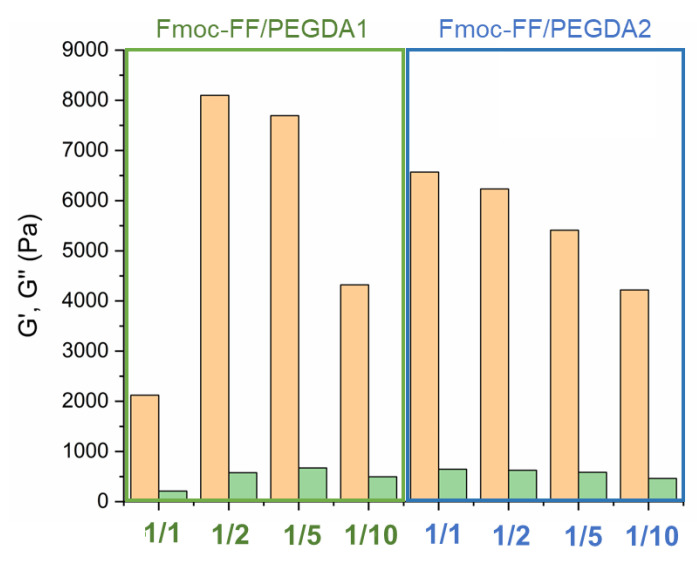
Rheological bar plots of 0.5 wt% mixed HGs. Plot reports both G’ (orange bar) and G” (green bar) moduli of each time sweep experiment (20 min, strain of 0.1%, frequency 1 Hz). Values are expressed on the Pascal (Pa) logarithmic scale.

Collectively, the linear viscoelastic region (LVE region) was found in the 0.02–3.0% stain range. The G’ and G’’ time sweep values, collected in the histograms of Figure 9 and Table 3, analytically demonstrate the gel state of all the tested matrices due to the values of G’ higher than G’’ and tan δ > 1 (G’/G’’> 1). All these values are higher than the ones for pure Fmoc-FF at the same concentration (G’~950 Pa), indicating that the multicomponent systems are characterized by enhanced mechanical properties.

This evidence has already been reported for other hybrid peptide/polymer or peptide/peptide matrices, suggesting the co-assembly and co-aggregation strategies as suitable methodologies to modify the mechanical performance of matrices [33,34].

In detail, it can be noted that PEGDA1-containing matrices result in greater rigidity than PEGDA2 ones at the same polymer ratio. This evidence is also pointed out by observing comparable strain break points (5 and 6% for PEGDA1 and PEGDA2, respectively) for systems at 1/10 ratios. In contrast, significant differences were found for the strain break points of PEGDA 1 and PEGDA2 at 1/1 ratios (61 and 12%, respectively).

All these data suggest a positive impact of the higher number of non-covalent interactions (H-bound) related to the number of PEG repetitions. According to thermodynamic principles, it is also suggested that there is a linear correlation between the modulus of rigidity and both the molecular weight and concentration of the polymer for pure PEG hydrogels. In the analysis of the Fmoc-FF/PEGDA series, a different trend can be detected for the two polymers. In the PEGDA1 series, G’ value is four times higher when passing from 1/1 to 1/2 ratios (2123 Pa to 8099 Pa). However, a further increase in the polymer does not cause an additional increase of the gel rigidity (7695 Pa for a 1/5 ratio). Finally, it can be observed a decrease in G’ for the gel at a 1/10 ratio (4323 Pa). This trend is also visible by comparing the tan δ values. On the contrary, a gradual and constant reduction of the G’ value is associated with the PEGDA2 series. Analogously, this decrease is also detectable in tan δ ratios. The general rheological behavior of Fmoc-FF/PEGDA hydrogels is not the expected one. Indeed, it was previously observed that an increase in the PEG concentration can allow an improvement in the mechanical modification of supramolecular entanglement. This evidence is indicative of a multifactorial correlation between the final G’ and the total intermolecular network interactions and physical parameters (including water mobility, the total hydrophilicity of the system, a progressive increase in the hydrophilicity for a higher PEGDA ratio, and predictable matrix rigidity).

The modulable mechanical responses of these matrices suggest good applicative versatility. The viscoelastic nature of the HGs recalls their potential employment as scaffold elements for tissue engineering or as tridimensional supporting materials for cell attachments. According to the G’ values, the proposed hydrogels are also candidates as reservoirs for the delivery of active pharmaceutical ingredients (APIs). The G’ values suggest suitable physical entrapment of host molecules and tunability in shape, thickness, and resistance. All these features are compatible with implant development. To further scrutinize this latter purpose, the capability of the resulting matrices to encapsulate and retain a drug was tested.

### 2.6. Release of Naphthol Yellow S

**Figure 10:**
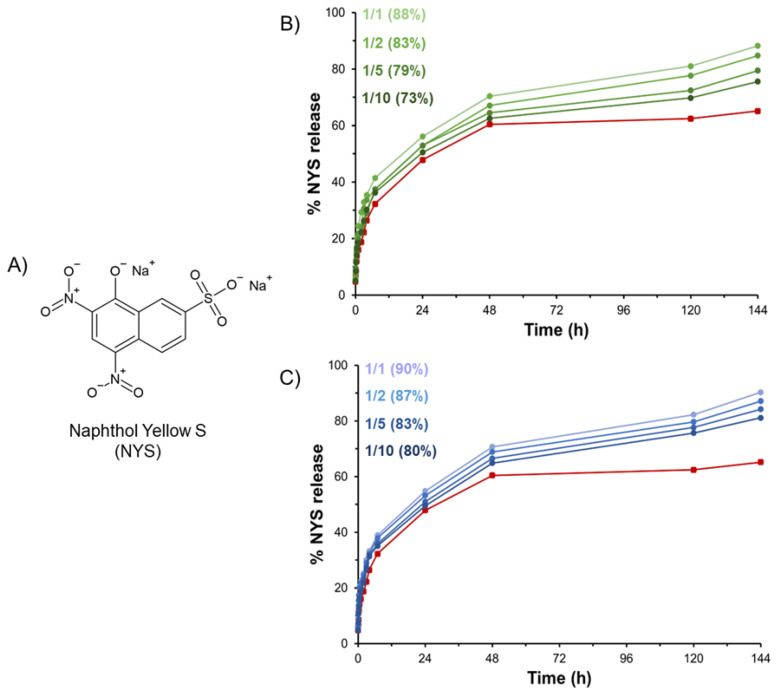
(**A**) Chemical structure of Naphthol Yellow S (NYS). Release profiles of NYS for Fmoc-FF/PEGDA1 (**B**) and for Fmoc-FF/PEGDA2 (**C**) hydrogels at different molar ratios. The NYS release from pure Fmoc-FF hydrogel is also reported for comparison (red line).

HGs are often proposed as matrices able to serve as drug reservoirs. Prolonged and modified release of active pharmaceutical ingredients, including small drugs or diagnostics, can be modulated via encapsulation in hydrogel matrices. According to this evidence, we attempted to load the hydrogels with Naphthol Yellow S (NYS), which is a water-soluble disodium salt of 5,7-dinitro-8-hydroxynaphthalene-2-sulfonic acid used as a histological dye and here employed as a drug model (Figure 10A). The hydrophilic nature of NYS allows for its incorporation it into the hydrogel during the rehydration step of the Fmoc-FF stock solution prepared in DMSO. The macroscopic observation of gels clearly indicates that the loading of the dye into the matrices does not affect the gelation process at the tested concentration (6.02 mmol·L^−1^). The amount of NYS encapsulated in the gel was considered to be 100% of the loaded dye. Release profiles of NYS from PEGDA-based HGs over time (up to 144 h) are reported in Figure 10 in terms of NYS released percentage.

From the inspection of Figure 10, it appears that all the mixed hydrogels exhibit a slower release with respect to pure Fmoc-FF HG (red line). This result can be explained as function of the higher swelling ratio q of hybrid hydrogels with respect to Fmoc-FF ones (see Table 1). Indeed, it is reasonable to expect the existence of a relationship between the passive water permeation of the hydrogel and the swelling ratio q, which is indicative of the mobile water in a completely swollen state. Moreover, the released percentage decreases from 88 to 73% and from 90 to 80% for PEGDA1 and PEGDA2, respectively, within the series by moving from a 1/1 to a 1/10 molar ratio. This trend suggests that the increase of PEGDA in the mix allows for higher retention of the NYS into the aqueous hydrogel matrix.

This result is not surprising considering the ability of PEGDA to establish non-covalent interactions such as hydrogen bonds with the NYS. According to this consideration, it is reasonable to expect that the percentage of the drug release decreases with the increase in the PEGDA amount and the PEGDA molecular weight.

## 3. Conclusions

Short and ultrashort peptides have been identified as manageable, tunable, and versatile building blocks for the fabrication of supramolecular systems. The features of peptides allow the combination, in the same system, of two or more molecular constituents, which can also differ in chemical characteristics. In the case of the study herein reported, for the first time, the development of mixed peptide-polymer matrices using Fmoc-FF and two different polydisperse diacrylate-capped PEGs (PEGDA, specifically PEGDA 575, PEGDA1, and PEGDA 250, PEGDA2) was evaluated. A solvent switch methodology was used for the hydrogel formulation, and different molar ratios of peptide/polymer (1/1, 1/2, 1/5, and 1/10 mol/mol) were evaluated. Both the polymer molecular weight and abundance were found to be able to modify the features of the hybrid materials in terms of water content, surface topology, stability, and model drug release (Napthol Yellow S) profiles. The supramolecular organization of the hydrogels is dictated by Fmoc-FF self-assembling, as supported by CD, FTIR, and WAXS analysis. Additionally, by simply modifying the quantity of the inserted polymer, a range of mechanically multivalent materials can be formulated, and each of them is easily adaptable to the desired application scope. The rheological analysis pointed out the versatility of the proposed matrices that, even if formed from the same chemical constituents, present different mechanical response. This evidence suggests the potential use of these materials in different application areas. Collectively, the data reported make possible a mutual comparison between the hybrid systems and the pure components, thus confirming the peptide-based approach as an easy, modulable, and accessible strategy for the design of novel nanostructured materials. The capability of these hydrogels to encapsulate NYS, as a very preliminary model drug, demonstrates the proof of concept that they can potentially serve as drug reservoirs for drug delivery applications. However, other important issues such as the hydrogel biocompatibility (at their different molar ratios) and the choice of the drug (in terms of net charge, lipophilicity, and molecular weight) have to be investigated before an in vivo application. Finally, the presence of diacrylate functionalities in the supramolecular Fmoc-FF hydrogel network could be employed to promote a photo-activated cross-linking reaction, thus providing the generation of interpenetrating networks (IPNs) with a higher organization level in the system.

## 4. Materials and Methods

*Material and Methods*: Fmoc-FF (in a lyophilized powder state) was purchased from Bachem (Bubendorf, Switzerland). Poly(ethylene glycol) diacrylate (PEGDA) with an average MW of 250 and 575 u.m.a. is commercially available from Merck (Bari, Italy). All the other chemicals are available from Fluka (Bucks, Switzerland), Carlo Erba (Emmendingen, Germany), or LabScan (Stillorgan, Ireland). They were used as received unless otherwise stated. UV-Vis measurements were carried out using a Thermo Fisher Scientific Inc. (Wilmington, Delaware, USA) Nanodrop 2000c, equipped with a 1.0 cm quartz cuvette (Hellma).

*Formulation of multicomponent hydrogels*: Hydrogels were prepared using the DMSO/H_2_O solvent switch methodology. Fmoc-FF was initially dissolved in DMSO at 100 mg/mL. HGs (a volume of 400 µL at a 0.5 wt% concentration) were obtained via hydration of 20 µL of the Fmoc-FF stock with 380 μL of PEGDA solutions. The metastable and opaque mixtures were vortexed (2 s) and aged at room temperature until the formation of a clear, self-supporting hydrogel. The efficiency of gel formation was macroscopically evaluated using the inverted tube test. The different Fmoc-FF/PEGDA molar ratios were obtained using several PEGDA solutions prepared in double distilled water (5.0 mL) by adding different amounts of pure polymers to water. ρ = 1.11 g/mL and ρ = 1.12 g/mL for PEGDA 250 (PEGDA2) and PEGDA 575 (PEGDA1), respectively.

*Swelling ratio (q) determination*: Hydrogel swelling ratios (*q*) were determined by adding a volume of 1.2 mL of doubly distilled water to each preformed matrix sample (0.50 wt%, V = 400 μL). The samples were then incubated overnight at room temperature. After this period of incubation, the water on top of the matrix was removed and the sample was weighted to collect the *Ws* value, which represents the weight of the swollen hydrogel. Then, the sample was freeze-dried and weighed again (*Wd*). The determined values were used in Equation (1) to calculate the gelling ratio, *q*.

(1) $$q=\left(\frac{Ws-Wd}{Ws}\right)\cdot 100$$

*Ringer’s solution stability test*: The degradation of the hydrogels was estimated according to the Ringer’s solution stability test, which consists in the estimation of the change in the hydrogel weight when 400 μL of gel (0.50 wt%) is incubated in an oven at 37 °C for 40 days with 1.2 mL of Ringer’s solution. This solution contains 12.9 mg of NaCl, 0.45 mg of KCl, and 0.48 mg of CaCl_2._ The experiment was performed in triplicate. The weight loss ratio (Δ*W*) was calculated as a percentage according to Equation (2):

(2) $$\Delta W=\left(1-\frac{Wt}{Wo}\right)\cdot 100$$

In which *Wo* and *Wt* are the weights of the hydrogel before and after the addition of the Ringer’s solution, respectively.

*Dehydration curve*: Dehydration profiles were acquired on 100 µL of each of the gel samples (0.5 wt%), located in a silicon mask on a glass slide. Each sample was initially weighed. Then, weight points were collected at: 0 min, 5 min, 10 min, 20 min, 30 min, 1 h, 2 h, 3 h, 4 h, 5 h, 6 h, 7 h, and 24 h. Dehydration was evaluated in terms of weight variation. The curve was reported as a residual weight percentage.

*Relaxometry:* NMRD (nuclear magnetic relaxation dispersion) profiles were measured on a SMARtracer fast-field-cycling relaxometer (Stelar S.n.c., Mede (PV), Italy) equipped with a Stelar VTC-91 for temperature control, from 0.01 to 10 MHz at 25 °C. Relaxation rates were measured at 16 different values of the applied magnetic field with an acquisition field of 7.2 MHz, a polarization field of 8.5 MHz, polarization time, and relaxation delay 4 times T1, 16 sampled delay times, and a switching time of 3 ms. Each NMRD profile was fitted according to the same model-free approach used in our previous investigation [27], similarly to that performed on other biomolecules in the presence of two distinct levels of motion (slow and fast) [65,66].

*Circular Dichroism (CD)*: Far-UV CD profiles were collected in the 350–190 nm wavelength range on a Jasco J-810 spectropolarimeter (equipped with a NesLab RTE111 thermal controller unit) and performed at 25 °C in a 0.1 mm quartz cell. A total of 150 µL of metastable mixtures (immediately after their generation) were kept undergoing gelation in cells. Other experimental settings used for the measurements are the following: sensitivity = 5 mdeg, scan speed = 20 nm·min^−1^ time constant = 16 s, bandwidth = 1 nm. Each spectrum was obtained by averaging three different scans. All the spectra are reported in optical density (mdeg/O.D.).

*Fourier-transform infrared spectroscopy (FTIR):* FTIR analysis was conducted on 400 µL of each gel (0.5 wt%) on a Jasco FT/IR 4100 spectrometer (Easton, MD) in the 400–4000 cm^−1^ acquisition range. The deconvolution of the amide I region was performed with built-in software. The spectra were collected in transmission mode and then converted into emission. Each sample was recorded with a total of 120 scans at a rate of 4 mm·s^−1^ against a KBr background.

*Xerogel Thioflavin T (ThT) assay:* the ThT assay was conducted on Fmoc-FF/PEGDA hydrogels in their xerogel forms. Macroscopic HGs were spread on a clean coverslip glass and dried overnight. The obtained samples were stained for 30 sec with 50 µL of a water solution of ThT (50 µmol·L^−1^). After removing the dye excess with filter paper, samples were dried overnight. Stained xerogels were imaged using a fluorescence Leica DFC320 video-camera (Leica, Milan, Italy) connected to a Leica DMRB microscope equipped with a 20 X objective and a Green Fluorescent Protein (GPF) filter. The software Image J (National Institutes of Health, Bethesda, MD) was used for analysis.

*Congo Red assay*: Congo Red (CR) assay was performed on samples both in solution and in the solid state. In solution, absorbance measurements of CR solution alone or mixed with hydrogels were recorded on a Nanodrop 2000c spectrophotometer equipped with a 1.0 cm quartz cuvette (Hellma). A stock solution of CR (3.5 mg in 500 μL) was freshly prepared in water and filtered through a 0.2 μm syringe. A total of 5 μL of this solution, 12-fold diluted, was added into 380 μL of each PEGDA solution used for hydration of the Fmoc-FF stock solution. The final CR concentration is 10 μmol·L^−1^. UV-Vis spectra of the samples were recorded between 400 and 700 nm at room temperature, and background was subtracted using a Congo Red spectrum in water as a reference solution. Xerogels, prepared on glass slides for deposition and air-drying of preformed hydrogels (~40 μL), were stained for a few seconds with a droplet (~5 μL) of a fresh CR solution, prepared as previously described [27]. Films were then observed under bright-field illumination and between crossed polars by using a Nikon AZ100 microscope.

*Scanning Electron Microscopy (SEM):* Fmoc-FF/PEGDA hydrogels were positioned on aluminum cover slips and left to air-dry at ambient conditions overnight. Then, the obtained xerogels were coated with Au film and imaged using a SEM (JEOL, Tokyo, Japan) operating at 20 kV.

*Wide-Angle and Small-Angle X-Ray scattering:* WAXS patterns were recorded from solid fibers prepared by the stretch frame method. An aliquot of ~20 μL of metastable, freshly prepared DMSO/H2O solutions (0.5 wt% final gel concentration) was placed between the ends of a wax-coated capillary (spaced 1.5 mm apart). The droplet was air-dried overnight, obtaining solid fibers [32]; 2D WAXS data were collected, as previously described, from the fibers at the X-ray MicroImaging Laboratory (XMI-L@b), equipped with an Fr-E + SuperBright rotating anode table-top microsource (Cu Kα, λ = 0.15405 nm, 2475 W), a multilayer focusing optics (Confocal Max-Flux; CMF 15-105), and a three-pinhole camera (Rigaku SMAX-3000) [67,68].

*Rheological characterization:* Rheological measurements were performed at 25 °C using a rotationally controlled stress rheometer (Malvern Kinexus) equipped with a 15.0 mm flat-plate geometry (PU20:PL61). A freshly prepared hydrogel sample (360 μL) at a concentration of 0.5 wt% was used for each experiment and located in a humidity chamber. A gap of 1.0 mm was used during measurements. Preliminary strain (0.01–100%) and oscillatory frequency (0.01–100 Hz) sweeps were conducted to identify the regime of linear viscoelasticity. Time-sweep oscillatory tests (in a 0.1% strain and 1.0 Hz frequency regime) were performed for 20 min. Final analyses are reported as G’(storage elastic modulus)/G” (shear loss or viscous modulus) ratio in Pascal [Pa].

*Naphthol yellow S encapsulation and release:* Naphthol yellow S (NYS) was encapsulated (at a final concentration of 6.02 mmol/L) in 400 µL of hybrid HGs, modifying the previously reported methodology. A solution of NYS, prepared in water, was analytically quantified via UV-Vis, using the molar extinction coefficient ε_430_ = 9922 L·cm^−1^·mol^−1^. ([NYS] = 0.012 mol·L^−1^). Then, 200 µL was added to properly prepare more concentrated solutions of PEGDA. These bicomponent solutions were used to rehydrate the Fmoc-FF DMSO stock in conical tubes (1.5 mL). On the top of the HGs, 800 µL of water was located. At well-defined time points, 400 µL of the top solution was replaced with an equal aliquot of water. The quantification of the NYS released from the hydrogels was assessed on the collected supernatants by UV-Vis spectroscopy (Abs at 430 nm). The amount of released dye was plotted as a percentage of the ratio between the amount released over time and the total loaded one. All the experiments were performed in triplicate.

## Figures and Tables

**Table 1:** Results of swelling and stability tests. The swelling ratio (*q*) and the weight loss ratio (Δ*W*) was calculated as a percentage according to Equations (1) and (2), respectively.

	Swelling Ratio (*q*)				Weight Loss Ratio (Δ*W*)			
Sample	1/1	1/2	1/5	1/10	1/1	1/2	1/5	1/10
Fmoc-FF/PEGDA 1	31.5	32.9	35.4	39.7	5.53	1.23	1.31	1.15
Fmoc-FF/PEGDA 2	30.1	31.2	33.1	36.1	4.47	3.01	2.85	2.09

**Table 2:** Parameters obtained through fitting of the ^1^H-NMRD profiles.

Sample	A_0_ (s^−1^)	β (s^−1^)	A_1_	τ_1_ (s)	A_2_	τ_2_ (s)	% slow	τ_C_^av^ (s)
Fmoc-FF	0.41	0.30	3.2 × 10^6^	1.3 × 10^−7^	2.6 × 10^5^	3.1 × 10^−6^	7.6	3.5 × 10^−7^
Fmoc-FF/PEGDA1 *1/1*	0.38	0.35	2.3 × 10^6^	7.8 × 10^−8^	6.5 × 10^4^	4.3 × 10^−6^	2.8	1.9 × 10^−7^
Fmoc-FF/PEGDA1 *1/5*	0.39	0.41	2.3 × 10^6^	5.2 × 10^−8^	7.5 × 10^4^	2.4 × 10^−6^	3.2	1.3 × 10^−7^
Fmoc-FF/PEGDA1 *1/10*	0.51	0.35	1.9 × 10^6^	9.3 × 10^−8^	5.5 × 10^6^	3.0 × 10^−6^	2.8	1.7 × 10^−7^
Fmoc-FF/PEGDA1 *1/20*	0.59	0.22	3.4 × 10^6^	1.8 × 10^−7^	1.6 × 10^5^	3.2 × 10^−6^	4.5	3.2 × 10^−7^
Fmoc-FF/PEGDA1 *1/30*	0.58	0.33	1.7 × 10^6^	1.6 × 10^−7^	1.0 × 10^5^	3.6 × 10^−6^	5.6	3.5 × 10^−7^
Fmoc-FF/PEGDA2 *1/1*	0.37	0.49	1.6 × 10^6^	4.5 × 10^−8^	4.7 × 10^4^	1.6 × 10^−6^	2.8	9.0 × 10^−8^
Fmoc-FF/PEGDA2 *1/5*	0.36	0.80	1.1 × 10^6^	4.5 × 10^−8^	4.3 × 10^4^	1.6 × 10^−6^	3.8	1.0 × 10^−7^
Fmoc-FF/PEGDA2 *1/10*	0.38	0.40	1.1 × 10^6^	2.0 × 10^−7^	4.2 × 10^4^	3.9 × 10^−6^	3.7	3.3 × 10^−7^

**Table 3:** Hydrogel rheological analysis. Reported data are storage modulus (G’), loss modulus (G’’) and tan δ (G’/G’’).

Sample	G’ (Pa)	G’’ (Pa)	tan δ
Fmoc-FF/PEGDA1 *1/1*	2123	210	10.1
Fmoc-FF/PEGDA1 *1/2*	8099	577	14.0
Fmoc-FF/PEGDA1 *1/5*	7695	672	11.5
Fmoc-FF/PEGDA1 *1/10*	4323	497	8.70
Fmoc-FF/PEGDA2 *1/1*	6569	646	10.2
Fmoc-FF/PEGDA2 *1/2*	6233	625	9.97
Fmoc-FF/PEGDA2 *1/5*	5411	586	9.23
Fmoc-FF/PEGDA2 *1/10*	4220	462	9.13

## Data Availability

Not applicable.

## Supplementary Materials

The following supporting information can be downloaded at: https://www.mdpi.com/article/10.3390/gels8120831/s1, Figure S1: Self-supporting hydrogel of Fmoc-FF/PEGDA1 1/50 mol/mol ratio; Figure S2: Water retention curve of mixed Fmoc-FF/PEGDA hydrogels. Figure S3: WAXS characterization of the mixed hydrogels Fmoc-FF: 2D WAXS data (on the left) and 1D WAXS meridional/equatorial profiles (on the right). Figure S4: Superimposition of 1D WAXS equatorial (A, B, C, D) and meridional (E, F, G, H) profiles for Fmoc-FF HG and PEGDA1 based HGs at the studied molar ratio. Figure S5: Superimposition of 1D WAXS equatorial (A, B, C, D) and meridional (E, F, G, H) profiles for Fmoc-FF HG and PEGDA2-based HGs at the studied molar ratio. Figure S6: Oscillation time sweeps tests for PEGDA1 1/1 (A) and PEGDA1 1/10 (C); frequency time sweeps tests for PEGDA1 1/1 (B) and PEGDA1 1/10 (D). Figure S7: Oscillation time sweeps tests for PEGDA2 1/1 (A) and PEGDA2 1/10 (C); frequency time sweeps tests for PEGDA2 1/1 (B) and PEGDA2 1/10 (D). Table S1: Meridional and equatorial peak positions in q (Å^–1^) and corresponding distance d = 2π/q (Å) of the mixed hydrogels; in red, the common peak with the Fmoc-FF reference fiber.
