# Supplementary material for: Incorporation of PEG Diacrylates (PEGDA) Generates Hybrid Fmoc-FF Hydrogel Matrices
Source: Gels. 2022 Dec 16;8(12):831. doi: 10.3390/gels8120831 (PMC9778368; doi:10.3390/gels8120831)
Supplement: Supplementary file 1 [file gels-08-00831-s001.zip › gels-2065346-supplementary.pdf]

Supporting Information

Incorporation of PEG diacrylates (PEGDA) generates hybrid Fmoc-FF matrices.

Elisabetta Rosa,[a],¥ Enrico Gallo,[b],¥ Teresa Sibillano,[c] Cinzia Giannini,[c] Serena Rizzuti,[d] Eliana Gianolio,[d] Pasqualina Liana Scognamiglio,[e] Giancarlo Morelli,[a] Antonella Accardo,[a] Carlo Diaferia[a],*

[a]Department of Pharmacy, Research Centre on Bioactive Peptides (CIRPeB), University of Naples "Federico II", Via Montesano 49, 80131 Naples (Italy)

[b]IRCCS Synlab SDN, Via E. Gianturco 113, 80143, Naples (Italy)

[c]Institute of Crystallography (IC), CNR, Via Amendola 122, 70126 Bari (Italy)

[d]Department of Molecular Biotechnologies and Health Science, University of Turin, Via Nizza 52, 10125, Turin (Italy)

[e] Department of Sciences, University of Basilicata, Via dell'Ateneo Lucano 10, 85100, Potenza, (Italy)

¥*These authors contributed equally*

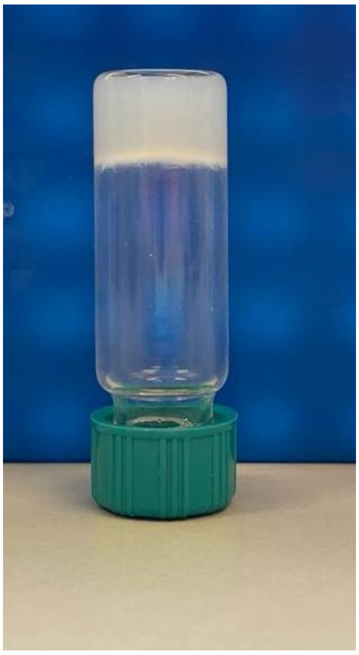

Figure S1: Self-supporting hydrogel of Fmoc-FF/PEGDA1 1/50 *mol/mol* ratio.

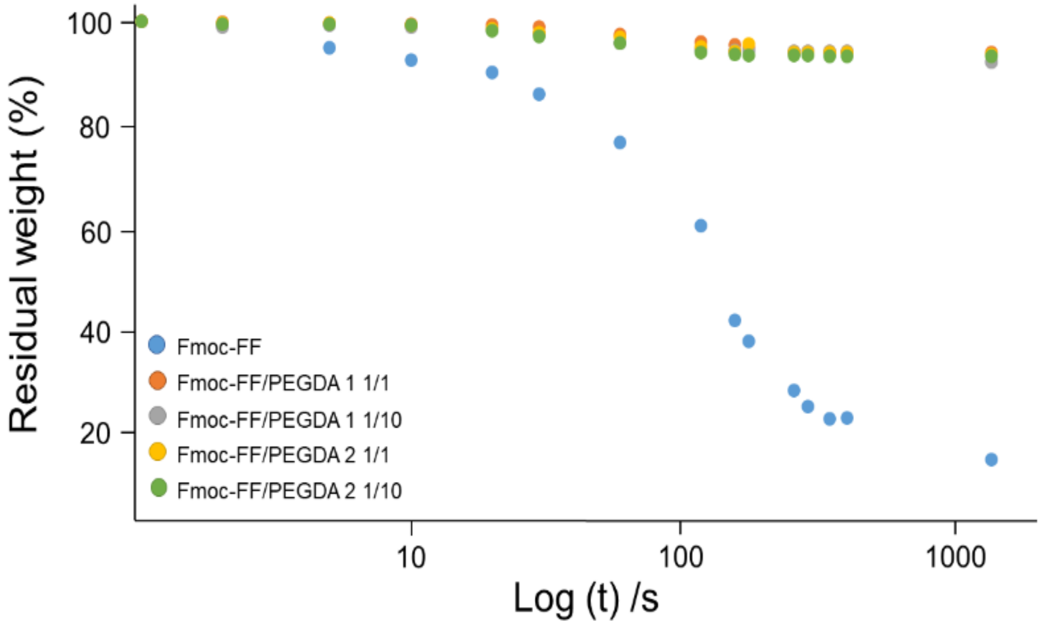

Figure S2: Water retention curve of mixed Fmoc-FF/PEGDA hydrogels.

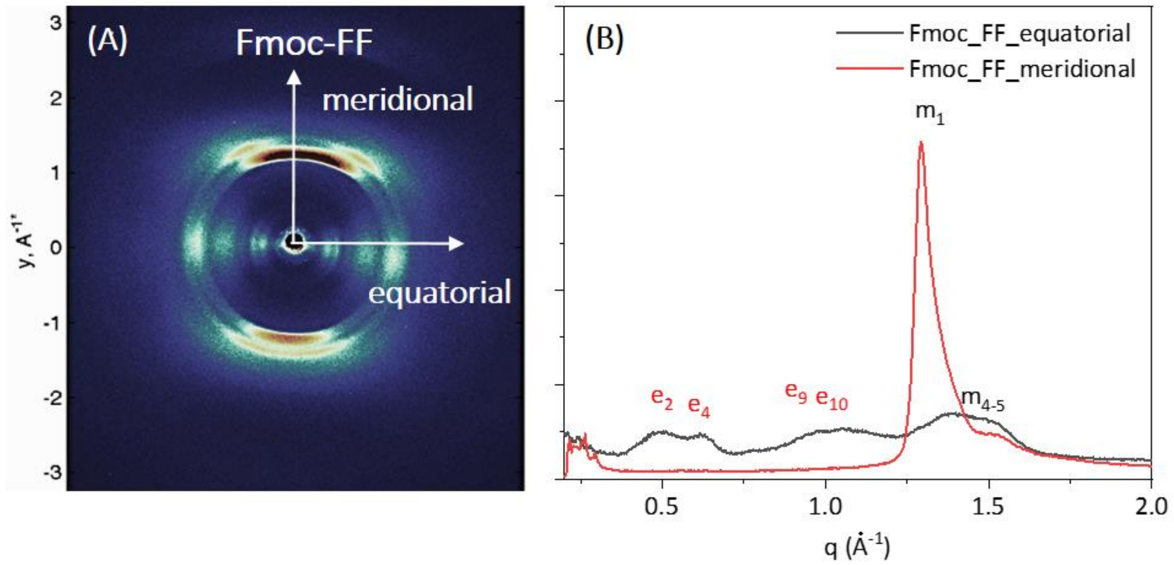

Figure S3: WAXS characterization of the mixed hydrogels Fmoc-FF: 2D WAXS data (on the left), and 1D WAXS meridional/equatorial profiles (on the right).

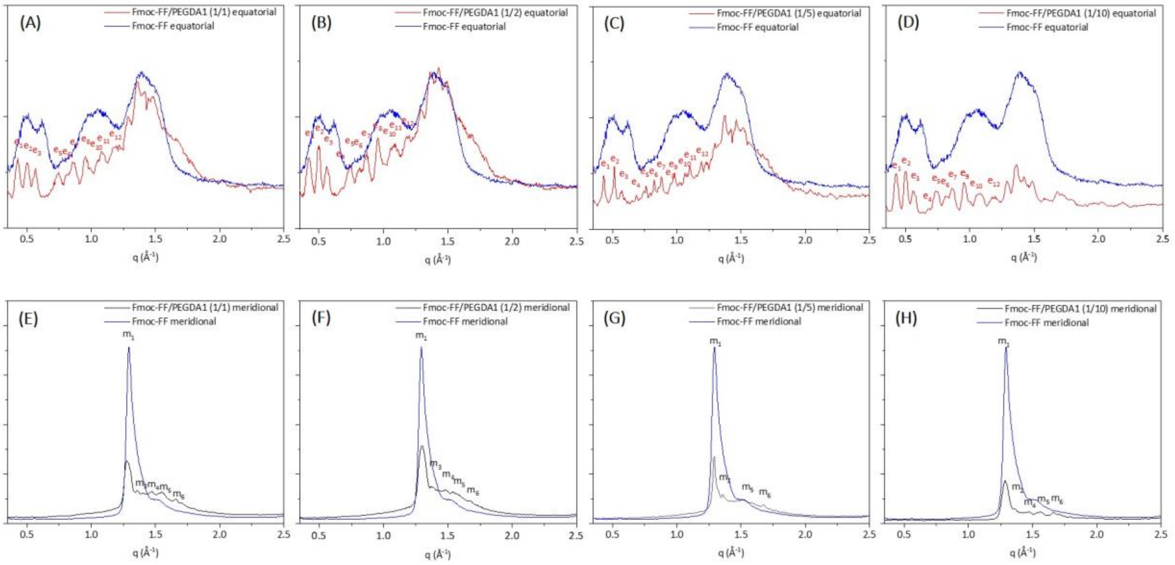

Figure S4: Superimposition of 1D WAXS equatorial (A, B, C, D) and meridional (E, F, G, H) profiles for Fmoc-FF HG and PEGDA1 based HGs at the studied molar ratio.

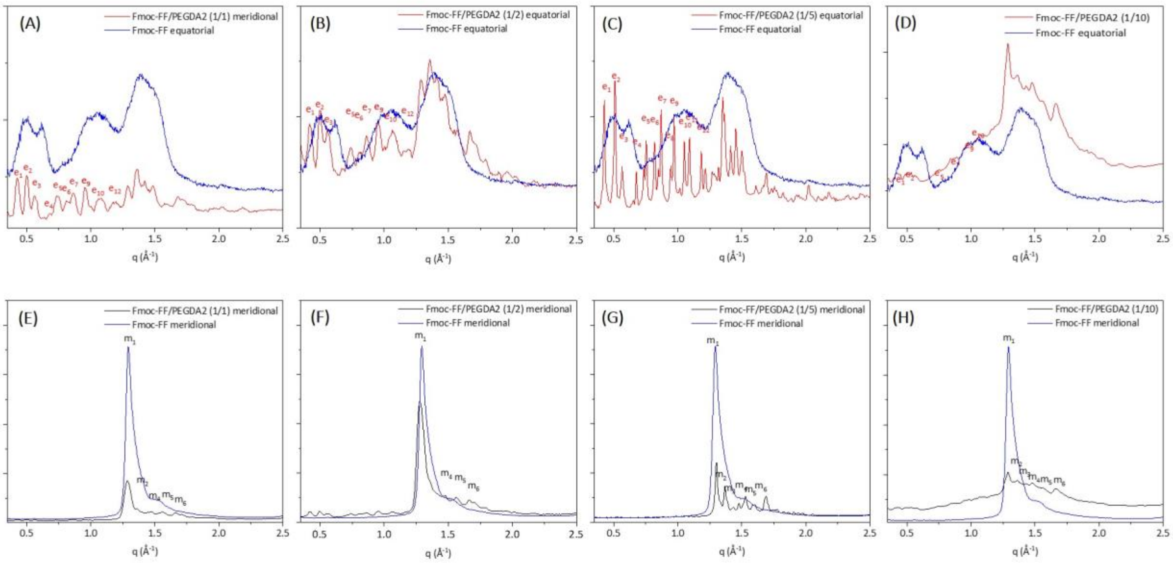

Figure S5: Superimposition of 1D WAXS equatorial (A, B, C, D) and meridional (E, F, G, H) profiles for Fmoc-FF HG and PEGDA2 based HGs at the studied molar ratio.

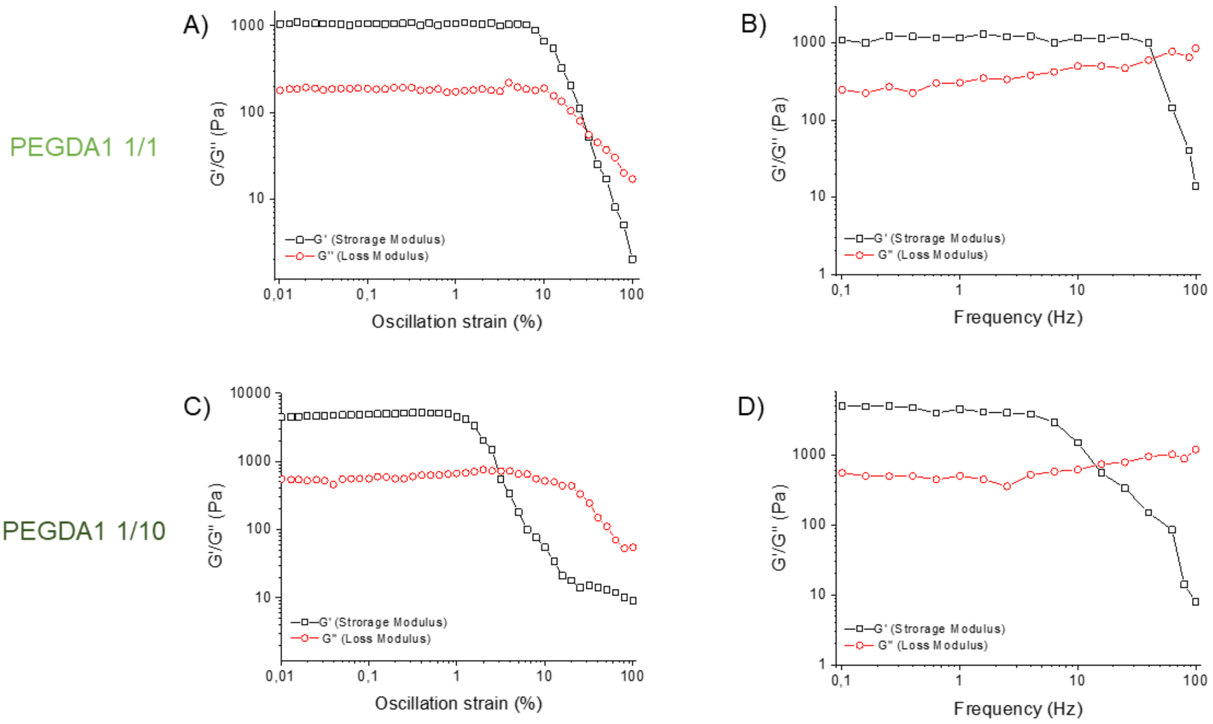

Figure S6: Oscillation time sweeps tests for PEGDA1 1/1 (A) and PEGDA1 1/10 (C); frequency time sweeps tests for PEGDA1 1/1 (B) and PEGDA1 1/10 (D).

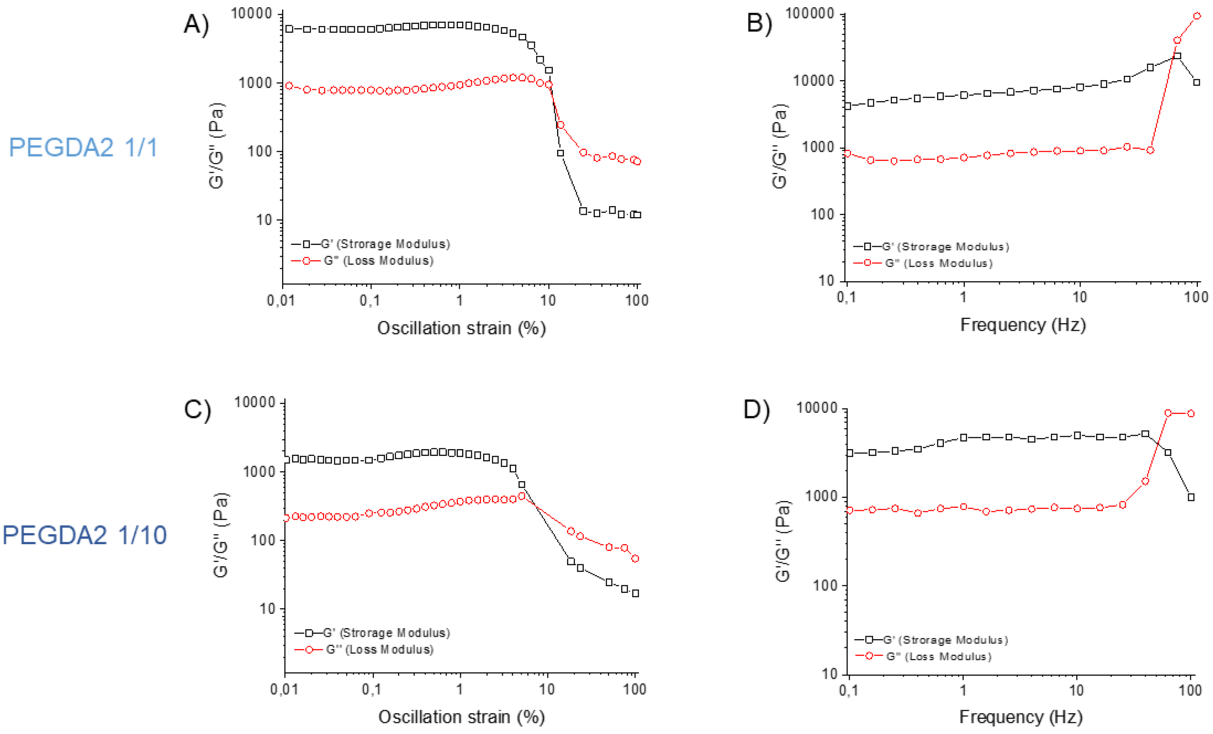

Figure S7: Oscillation time sweeps tests for PEGDA2 1/1 (A) and PEGDA2 1/10 (C); frequency time sweeps tests for PEGDA2 1/1 (B) and PEGDA2 1/10 (D).

Table S1: Meridional and equatorial peak positions in q (Å^{-1}) and corresponding distance d = 2π/q (Å) of the mixed hydrogels; in red the common peak with the Fmoc-FF reference fiber.

**Reflections**	q [Å^{-1}] ± 0.02	d [Å] ± 0.5
**Equatorial**	e_1 = 0.42	d_{e1}=14.96
	**e_2 = 0.50**	**d_{e2}=12.57**
	e_3 = 0.56	d_{e3}=11.22
	**e_4 = 0.65**	**d_{e4}=9.67**
	e_5 = 0.74	d_{e5}=8.49
	e_6 = 0.81	d_{e6}=7.76
	e_7 = 0.87	d_{e7}=7.22
	e_8 = 0.94	d_{e8}=6.68
	**e_9 = 0.97**	**d_{e9}=6.48**
	**e_{10} = 1.05**	**d_{e10}=5.98**
	e_{11} = 1.09	d_{e11}=5.76
	e_{12} = 1.18	d_{e12}=5.32
**Meridional**	**m_1 = 1.29**	**d_{m1}=4.91**
	m_2 = 1.32	d_{m2}=4.76
	m_3 = 1.42	d_{m3}=4.42
	**m_4 = 1.46**	**d_{m4}=4.30**
	**m_5 = 1.54**	**d_{m5}=4.08**
	m_6 = 1.62	d_{m6}=3.88
